# Measuring Psychological Well-Being and Behaviors Using Smartphone-Based Digital Phenotyping: An Intensive Longitudinal Observational mHealth Pilot Study Embedded in a Prospective Cohort of Women

**DOI:** 10.2196/71375

**Published:** 2025-09-03

**Authors:** Li Yi, Claudia Trudel-Fitzgerald, Cindy R Hu, Grete Wilt, Jorge Chavarro, Jukka-Pekka Onnela, Francine Grodstein, Laura D Kubzansky, Peter James

**Affiliations:** 1 Department of Population Medicine, Harvard Medical School and Harvard Pilgrim Health Care Institute, 401 Park Drive, Suite 401 East, Boston, MA, 02215, United States; 2 Department of Nutrition, Harvard TH Chan School of Public Health, Boston, MA, United States; 3 Département of Psychology, Université du Québec À Trois-Rivières, Trois-Rivières, QC, Canada; 4 Research Center, Universitaire en Santé Mentale de Montréal, Montréal, QC, Canada; 5 Lee Kum Sheung Center for Health and Happiness, Harvard University, Boston, MA, United States; 6 Department of Environmental Health, Harvard TH Chan School of Public Health, Boston, MA, United States; 7 Channing Division of Network Medicine, Department of Medicine, Brigham and Women's Hospital and Harvard Medical School, Boston, MA, United States; 8 Department of Biostatistics, Harvard TH Chan School of Public Health, Boston, MA, United States; 9 Rush Alzheimer's Disease Center, Rush University Medical Center, Chicago, IL, United States; 10 Department of Social and Behavioral Sciences, Harvard TH Chan School of Public Health, Boston, MA, United States; 11 Department of Public Health Sciences, University of California, Davis School of Medicine, Medical Sciences 1-C, One Shields Ave, Davis, CA, 95616, United States, 1 530-752-5676

**Keywords:** daily mobility, digital phenotyping, ecological momentary assessments, epidemiological cohort, health behavior, psychological well-being, happiness, smartphone apps and sensors, mobile phone

## Abstract

**Background:**

Intensive measures of well-being and behaviors in large epidemiologic cohorts have the potential to enhance health research in these areas. Yet, little is known regarding the feasibility of using mobile technology to collect intensive data in the “natural” environment in the context of ongoing large cohort studies.

**Objective:**

We examined the feasibility of using smartphone digital phenotyping to collect highly resolved psychological and behavioral data from participants in a pilot study with participants in Nurses’ Health Study II, a nationwide prospective cohort of women.

**Methods:**

In this pilot study, an 8-day intensive smartphone protocol was implemented using the “Beiwe” smartphone app. Participants (n=181) completed a baseline survey on day 1 and answered ecological momentary assessment (EMA) surveys twice daily (days 2-8; early afternoon and evening) using their smartphone and provided minute-level accelerometer and GPS data. A feedback survey at the end of Substudy queried participants’ experience with the app and data collection process. We assessed adherence to the protocol by examining completion on EMA surveys and completeness of accelerometer and GPS data at the participant, participant day, and prompt levels.

**Results:**

Our pilot study demonstrated modest overall compliance with smartphone-based surveys: the baseline survey completion rate was high (156/181, 86.2%), but average daily EMA response rates during the 7-day period were lower with 55.6% (SD 3.9%) for early afternoon and 54.7% (SD 3.2%) for evening. We also observed good average daily completeness of smartphone accelerometer (mean 62.0%, SD 4.5%) data and GPS data (mean 57.7%, SD 3.1%). The feedback survey revealed that the participants found “the app easy to use” (median 85.0 on a scale of 1‐100) and were “willing to repeat similar studies” (median 85.0 on a scale of 1‐100). Although participants reported feeling their participation was a positive experience (median 64.0 on a scale of 1‐100), they also identified some important issues, including user fatigue due to repetitive daily surveys.

**Conclusions:**

We observed modest compliance with smartphone surveys and completeness of smartphone passive sensing data in this pilot study compared with similar studies in the past. However, this was not unexpected, given our participants were older (aged 57‐75 years, with more than 3 decades of follow-up at the time of the substudy) and may encounter more technological barriers, not to mention that the indication of willingness to participate in such studies again was fairly high. Our findings also highlight that the success and data quality of efforts to obtain daily measures may vary depending on data type and emphasize the need to improve the design of the EMA survey to improve or sustain participant engagement over the study period. Overall, our findings suggest smartphone-based digital phenotyping as a promising technology when embedding in large epidemiological cohorts to collect intensive longitudinal observation data.

## Introduction

### Background

Many dimensions of psychological well-being—both stable traits (eg, optimism) and time-varying states (eg, happiness)—are linked to positive physical health outcomes such as a lower risk of chronic disease and mortality [1-3]. Health behaviors, including diet, physical activity, and sleep, are risk factors for many chronic diseases and may also be influenced by psychological well-being, thus acting as pathways by which psychological well-being affects physical health [4-9]. However, most studies have collected psychological and behavioral data annually or at longer intervals, limiting the ability to examine dynamic relationships between the two, which may be critical for elucidating the mechanisms linking psychological well-being to chronic disease. To better understand the relationships between psychological well-being and health behaviors, it is necessary to collect granular data on both factors on a daily or diurnal basis [9,10].

To address this limitation, emerging research has focused on the use of smartphone-based digital phenotyping, defined as the moment-by-moment quantification of individual human phenotypes via personal smartphone data, to collect temporally intensive longitudinal (eg, minute-by-minute) data [11]. In particular, ecological momentary assessment (EMA) can be combined with accelerometer-enabled smartphones to measure participants’ behaviors and experiences repeatedly in real time. This approach facilitates efficient and cost-effective digital phenotyping of psychological well-being and specific health behaviors in a naturalistic setting [12-16]. Consequently, EMA conducted with personal smartphones may facilitate large-scale data collection to study psychological well-being and behavioral health with finer temporal resolution than is possible with standard survey research. Recruiting samples for such research can be difficult and time-consuming, whereas drawing on existing cohorts with participants who have a history of compliance with and commitment to the overall study may provide a more efficient means for conducting such studies. However, embedding smartphone digital phenotyping in large epidemiological cohorts has not yet been sufficiently explored [17], nor has the acceptability and effectiveness of this strategy among populations that have generally agreed to participate in survey research been tested.

### Objectives

This study describes the methodology for integrating the Beiwe smartphone digital phenotyping platform into the Nurses’ Health Study II (NHSII), a large and long-running US-based cohort of middle-aged and older women (aged 55‐77 years). We collected data on psychological well-being, physical activity, and geolocation from 181 NHSII participants during an 8-day intensive measurement burst that included daily EMA surveys and minute-level accelerometer and GPS sensing. Our aim was to assess the feasibility of using smartphone digital phenotyping to collect highly resolved psychological, behavioral, and environmental data by examining compliance with the survey schedule, completeness of passive sensing data, and participant responses to the end-of-study feedback survey.

## Methods

### Beiwe Smartphone Substudy Embedded Within the NHSII

NHSII is an ongoing prospective cohort study involving 116,429 female registered nurses aged 25‐42 years at enrollment in 1989. The participants currently reside in all states of the United States. Participants completed a mailed questionnaire asking detailed questions about demographic characteristics, lifestyles, and history of chronic diseases at the time of enrollment. Participants are invited to complete biennial questionnaires to update their medical status and lifestyle factors [18]. The Beiwe Smartphone Substudy of NHSII is a pilot study designed to examine the feasibility of using smartphone apps to collect psychological well-being, physical activity, and geolocation data at a granular level (eg, daily and minute-by-minute) among participants in this ongoing cohort study. The substudy targeted those who have most frequently engaged with the larger NHSII through biennial survey completion and provision of biological specimens [12]. For this pilot study, participants took part in an 8-day intensive measurement burst via the Beiwe app, completing psychological well-being surveys twice daily while the app collected smartphone accelerometer and GPS data at the minute level. At the beginning of the study, the participants were instructed to download the Beiwe app on their smartphones and log on via provided credentials. During the 8-day data collection period, participants were asked to keep their smartphone on them during waking hours, charge it regularly, and connect to Wi-Fi at least once a week to synchronize their data. Trained research assistants provided technical support throughout the study by phone or email. Upon completion of the study, participants were instructed to delete the app, and their user accounts were deactivated. The pilot study ran from July 21, 2021, to November 21, 2021 (the last day the last participant provided data within 8-day period).

Our goal was to recruit 200 participants for the substudy. To be eligible for an invitation to the pilot study, participants must have been active NHSII participants and not participating in other NHSII substudies. From this pool, we randomly selected 600 individuals to whom we emailed invitations to complete an eligibility screener. The screener included the following additional eligibility criteria: (1) owns an Apple or Android smartphone, (2) lives in the contiguous United States (excluding Hawaii, Alaska, or Puerto Rico), and (3) has access to Wi-Fi at least once a week. Upon confirmation of eligibility, there was an informed consent process; after consenting, they were instructed to download the Beiwe app from the iOS or Android app stores.

### Data Collection—Beiwe App

The Beiwe app guided participants through onboarding, during which they gave their consent for the app to assess location data and to send notifications (see Multimedia Appendix 1, eg, screenshots of the app as viewed by the participant). Upon first opening the app (day 1), participants completed a baseline survey and then received 14 EMA surveys over the next 7 days (days 2-8), with 2 surveys administered daily during the same time windows each day. The shorter early afternoon survey (21 questions, including some branched yes or no questions) was sent out randomly between 12 PM and 3 PM; the longer evening survey (63 questions, including some branched yes or no questions) was sent out at 6 PM. The app notified participants when a survey was available for completion and marked it as incomplete if it was not completed before the next survey was sent. For each completed survey, the app recorded the start and end time and computed the duration. Meanwhile, the Beiwe app was programmed to collect raw GPS data every 15 minutes for 90 consecutive seconds and raw accelerometer data at 10 Hz every 30 seconds for 10 consecutive seconds. This approach was intended to minimize battery consumption while maintaining the ability to determine participant movement patterns and behavior throughout the day [19]. Technical support was available throughout the duration of the substudy for participants who encountered issues downloading or using the app. At the conclusion of our study, we downloaded the study data via the “Mano” algorithm in the “Forest” Python library (Python, version 3.9.2; Python Software Foundation).

### Smartphone-Based Measures

We assessed our key variables of interest and others that could be relevant when studying linkages between psychological well-being and behavior. We used a standard set of self-report items included on the baseline and EMA surveys administered twice daily (Multimedia Appendix 2), as well as passive sensing of smartphone accelerometer and GPS data.

#### Psychological Well-Being

In the baseline survey, we assessed *dispositional optimism*, a stable attribute positively associated with higher psychological well-being using the validated Life Orientation Test—Revised [20]. Participants rated their agreement with 3 positive and 3 negative statements on a 5-point Likert scale ranging from “Strongly disagree” (0) to “Strongly agree” (4). The positive statements included the following: (1) In uncertain times, I usually expect the best. (2) I’m always optimistic about my future. (3) Overall, I expect more good things to happen to me than bad. The negative statements included the following: (1) If something can go wrong for me, it will. (2) I hardly ever expect things to go my way. (3) I rarely count on good things happening to me. An overall optimism score was calculated by summing the positive and reverse scores of the negative statements [20].

We assessed time-varying psychological states contemporaneously (in the early afternoon EMA survey) and retrospectively (in the evening EMA survey) using the modified version of the Positive and Negative Affect Schedule—Expanded Form (PANAS-X) [21]. The 10 PANAS-X items designed to capture momentary affect included in the early afternoon EMA surveys were queried using the stem “Right now, I feel….” with response options yes or no scored as 1 or 0, respectively. Four items (happy, determined, enthusiastic, and curious) were summed to derive a *positive affect* scale, and 6 items (sad, afraid, angry, lonely, nervous, and worried) were summed to derive a *negative affect* scale.

A modified PANAS-X designed to capture affect experienced over the course of the day was administered in evening EMA survey and included 11 items queried using the stem “Today; did you feel….” with response options yes (score = 1) or no (score = 0). Five items are summed to derive a scale capturing *positive affect in the day* (happy, determined, enthusiastic, curious, and grateful) and 6 items are summed to derive a scale capturing *negative affect in the day* (sad, afraid, angry, lonely, nervous, and worried). In addition, participants were asked to report when they experienced these feelings (midnight: 5:59 AM; 6-11:59 AM; noon: 5:59 PM; 6-11:59 PM). The evening EMA also included a question on *life satisfaction*: “How satisfied are you with your life at this moment?” (queried on a scale of 1 [very unsatisfied] to 5 [very satisfied]) [22].

#### Health Behaviors

We obtained participants’ *physical activity* (ie, walking steps and walking time) by processing raw accelerometer data with the “Oak” walking recognition method in the “Forest” Python library (Python, version 3.9.2), which estimates gait cadence per second. We have focused on walking because it is the most common form of physical activity performed daily by middle-aged and older-aged women and has been associated with cognitive function and disease outcomes such as cardiovascular disease and cancer [23,24]. Our walking recognition method has been validated against 20 publicly available annotated datasets on walking activity data collected at various body locations (thigh, waist, chest, arm, and wrist). It estimates walking periods with high sensitivity and specificity and is able to account for missing data due to noncontinuous sampling to conserve smartphone battery [25]. Due to the large volume of raw accelerometer data, we implemented an “on-the-cloud” processing approach. Specifically, we fetched hourly level raw data, processed them in temporary cloud computing memory by applying the “Oak” algorithm, and stored the processed results (minute-level step counts) locally without downloading raw data. This approach substantially reduced the processing time. Details of the validated walking detection algorithm for Beiwe are available in a previous study [25] and through the GitHub link [26]. After identifying the walking periods, we aggregated the second-level data to the minute level, calculating the total number of seconds walked and step counts per minute.

Participants also reported their previous night’s sleep and wake times in the evening EMA survey, which we used to determine their *daily sleep duration*. Additionally, they reported their *sleep quality* during the previous night. Multimedia Appendix 2 lists the answer choices for the sleep survey.

#### Additional Measures

We assessed a number of key covariates relevant to the association between psychological well-being and health behaviors. First, since psychological distress can influence behaviors [13,27] and is related to facets of psychological well-being, we measured depressive symptoms using the Center for Epidemiologic Studies Depression Scale [28] in the baseline survey. Second, we included items to assess strategies participants may use to regulate their affective states (ie, emotion regulation and coping strategies) in the context of stressful events or positive experiences since these may also be related to behaviors [29-32]. These were included on both daily EMA surveys. In the early afternoon EMA survey, we queried the use of strategies to regulate emotions in the context of stressful events occurring in the past hour [33]. Items assessed the nature of the stressful event (eg, argument, financial issues, and traffic) and stress levels (“not at all” or “a little” or “somewhat” or “very”). Participants then rated their levels of agreement on a 5-point Likert scale (strongly disagree to strongly agree) with a list of strategies they might have used to cope with such events (eg, “I controlled my emotions by not showing them” and “I made a plan to make the situation better”). In the evening EMA survey, we presented participants with a list of stressful events and positive experiences and asked whether any had occurred in the past 24 hours (yes or no) and also queried levels of stressful or positive experience on a 4-point Likert scale of 1 (not at all), 2 (a little), 3 (somewhat), and 4 (very) in relation to each event (Multimedia Appendix 2). Additionally, in the evening survey, participants reported which positive and negative emotion they experienced most intensely that day (eg, happy and sad). They were then asked to indicate on a 5-point Likert scale (strongly disagree to strongly agree; Multimedia Appendix 2) the extent to which they used each strategy to cope with such events from a list of strategies presented (eg, “I reveled in the moment and concentrated on how good I felt” and “I made a plan to make the situation better”) [33]. They were also asked whether they changed their diet in response to the indicated emotion (ie, increased/decreased/did not change how much I ate) and whether this change was a way to comfort themselves with response options of yes or no. Finally, we inquired about psychotropic medication use in our evening EMA survey, asking whether participants had taken any of the following medications in the prior day: antidepressant medication (for any reason), antianxiety medication, and sleep medication.

Considering that individual daily mobility and environmental contexts may also be important [34], we obtained participants’ geolocation every minute by processing raw smartphone GPS data using a Python-based method called “Jasmine” in the “Forest” library (Python, version 3.9.2). This algorithm imputes minute-level geolocation and also calculates daily summary statistics measuring participants’ mobility, such as time spent at home and on trips and the number of places visited. Details of the validated GPS data-processing algorithm for Beiwe are available in a previous study [35] and through the GitHub link [26].

#### End-of-Study Feedback Survey

We invited participants to complete a feedback survey at the conclusion of the study to evaluate their overall experience in participating in the pilot study and their willingness to engage in any future smartphone-based data collection. This survey included items querying their overall experience, any difficulties using the app, and their likelihood of participating in similar future studies and offered the opportunity to provide open-ended feedback on app design and study experience. Feedback survey questions are shown in Multimedia Appendix 2.

#### Sociodemographic, Lifestyle, and Health Characteristics

To evaluate potential differences between study participants and those in the parent study who did not participate, we also extracted key sociodemographic and health characteristics from the baseline NHSII surveys. These included factors that are not time-varying (race) and time-varying ones from the 2019‐2021 NHSII biennial questionnaire, closest in time to the start of the pilot study in August 2021. These include participants’ age at the time of the substudy, race (White/Black/Other; non–time-varying), husband’s level of educational attainment (high school or less/more than high school, reported in 1999), percentages married or in a relationship, smoking status (current/former/never), BMI (calculated as weight in kilograms divided by height in meters squared), Alternative Healthy Eating Index (AHEI) diet score (continuous scores 0‐100) [36], and region of residence (Northeast/Midwest/South/West) based on 2010 Census data [37]. The AHEI assigns ratings to foods and nutrients predictive of chronic disease [36]. The AHEI is created by a group of researchers at Harvard TH Chan School of Public Health as an alternative to the US Department of Agriculture’s Healthy Eating Index, which measures adherence to the federal Dietary Guidelines for Americans.

### Statistical Analysis

Our examination of adherence to the smartphone digital phenotyping data collection protocol had 2 components. For the smartphone EMA surveys, we assessed compliance at the time of receiving each EMA prompt at participant day and participant levels. At the *participant level*, we calculated separate response rates for the baseline survey (day 1) and for the daily early afternoon and evening EMA surveys (days 2-8 only), each defined as the total number of surveys submitted by all participants out of all possible EMA prompts. We also calculated the proportions of participants with 25% (at least 2 out of 7 surveys), 50% (at least 4 out of 7 surveys), and 75% (at least 6 out of 7 surveys) response rates for the early afternoon and evening EMA surveys separately. At the *participant day* level, we examined the daily response rates for early afternoon and evening EMA surveys separately from days 2 to 8 (defined as the total number of surveys submitted by all participants in days 2, 3, …, 8 divided by total number of participants). In addition, we summarized the length of time it took to complete a given survey (ie, completion duration) in minutes for the baseline survey and for each daily EMA assessment.

To assess the completeness of passive sensing data (accelerometer and GPS), we identified days with high-quality data (ie, valid days) as those with at least 600 valid minutes (10 valid hours) per day, following the established criteria in GPS-based physical activity studies [34]. We also define noncollection days as days without data. In this study, the primary reason for noncollection was attrition (ie, the app was deleted from a participant’s phone before the 7-day study period because the participant herself unenrolled from the substudy). Another reason was sensor noncollection, which could be due to a participant forgetting to charge their phone or disabling the GPS or a major update from the operating system causing the Beiwe app to malfunction temporarily, among other reasons. A valid minute was defined as one with at least 1 second of observation time, following guidelines provided by previous Beiwe data studies [25]. We then calculated data completeness at both the *participant day* and *participant* levels. At the *participant day* level, we summarized the valid hours per day for all participants and days and calculated the percentages of valid, nonvalid, and noncollection days, respectively. At the *participant* level, data completeness was defined as the percentage of valid days out of 7 possible days (days 2-8), with proportions calculated for participants with 25% (minimum of 2 valid days), 50% (minimum of 4 valid days), and 75% (minimum of 6 valid days) completeness of accelerometer and GPS data. To test the sensitivity of this measure of completeness to an alternative threshold, we applied a stricter criterion defining a valid day as one with at least 960 valid minutes (16 valid hours), accounting for typical 8-hour sleep periods. Some participants did not delete the app at the conclusion of the study (ie, after day 8) and left the app running for up to 150 days, resulting in the collection of additional EMA surveys and passive sensing data. Their data beyond the study period were not included in the “Smartphone Data Characteristics” section. All statistical analyses were conducted using R (version 4.3.3; R Core Team).

### Ethical Considerations

All procedures performed in studies involving human participants were in accordance with the ethical standards of the institutional review boards of the Brigham and Women’s Hospital (protocol numbers 1999P003389). All participants provided informed consent for data collection at the beginning of the pilot study. Participants were not compensated. We encrypted raw data collected by the Beiwe app before sending them to the Amazon Web Services production server. Subsequently, we either securely transferred data to the analytical server (for GPS and survey data) or deleted those data from the temporary storage on the analytics server after processing (for accelerometer data). We protected Amazon Web Services production and analytics servers by 2-factor authentication and Secure Shell Protocol keys. To protect participant identity, we did not enter personal or health-related information through Beiwe.

## Results

### Participant Characteristics

**Figure 1.**
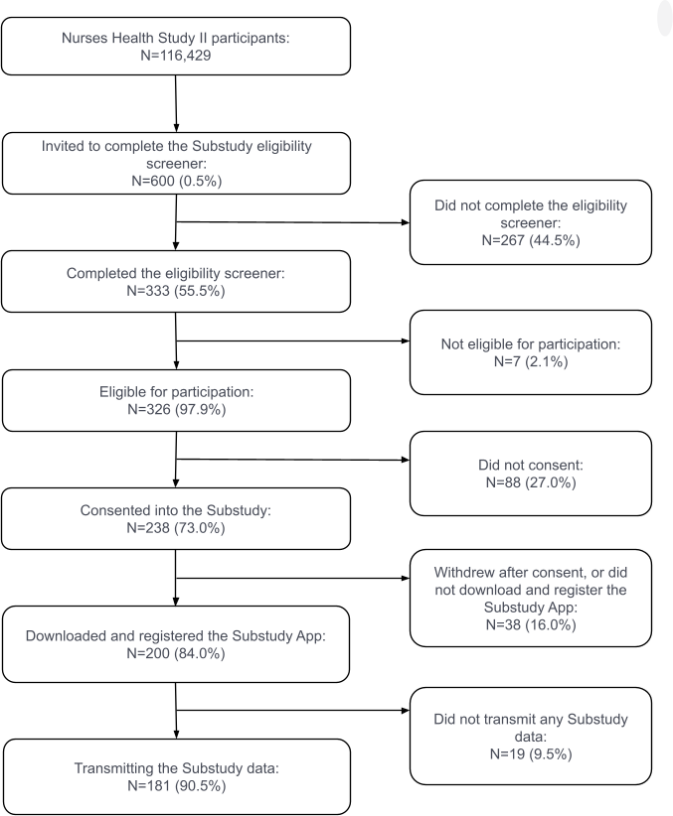
A CONSORT (Consolidated Standards of Reporting Trials) diagram of participation numbers at different recruitment stages of the Beiwe Smartphone Substudy of Nurses’ Health Study II.

We invited 600 NHSII participants (600/116,429, 0.52% of the larger NHSII cohort) to complete the eligibility screener. Of these 600 participants, 333 (55.5%) completed the screener, 326 (54.3%) were eligible to participate, 238 (39.7%) consented, 200 (33.3%) downloaded and registered the app, and 181 (30.2%) transmitted data. A CONSORT (Consolidated Standards of Reporting Trials) diagram of the participants at different recruitment stages is shown in Figure 1. Table 1 compares the demographics of the Beiwe pilot study participants who transmitted data against those who consented, who were invited, and the active NHSII participants at the beginning of the pilot study (late July 2021). Overall, our sample had a mean age of 67.8 (SD 3.9) years, were all female, and predominantly White (178/181, 98.3%). Most participants (148/181, 81.8%) were married and 58.6% (106/181) were never smokers. Breaking down by Census regions, 11.6% (21/181) of the participants lived in the Northeast, 17.7% (32/181) lived in the West, 26.0% (47/181) lived in the South, and 44.8% (81/181) lived in the Midwest. Most participants (84.5%, 153/181) were iOS users. Across the variables we considered, there were no major differences among the invited, consented, and enrolled participants. Additionally, no major demographic differences were observed between iOS and Android users (Multimedia Appendix 3).

**Table 1.** Characteristics of active Nurses’ Health Study II participants at the beginning of the Beiwe smartphone pilot study (August 2021) who were invited, consented, and participated in the study.

Variable	Beiwe Substudy participants—invited (n=600)	Beiwe Substudy participants—consented (n=238)	Beiwe Substudy participants—registered and downloaded the app (n=181)
Age (years), mean (SD)	68.2 (4.1)	68.0 (4.0)	67.8 (3.9)
Race, n (%)			
White	590 (98.3)	235 (98.7)	178 (98.3)
Black	0 (0.0)	0 (0.0)	0 (0.0)
Other/more than 1 race	10 (1.7)	3 (1.3)	3 (1.7)
Husband’s highest level of education, n (%)			
High school or less	160 (26.7)	70 (29.4)	51 (28.2)
More than high school	440 (73.3)	168 (70.6)	130 (71.8)
Marital or in a relationship, n (%)			
Married or in a relationship	491 (81.8)	190 (79.8)	148 (81.8)
Smoking status, n (%)			
Current	13 (2.2)	5 (2.1)	5 (2.8)
Former	195 (32.5)	91 (38.2)	70 (38.7)
Never	392 (65.3)	142 (59.7)	106 (58.6)
BMI (kg/m^2^), mean (SD)	26.8 (6.2)	27.0 (6.0)	27.0 (6.4)
AHEI score^a^, mean (SD)	69.0 (12.7)	70.2 (12.7)	70.0 (12.4)
Region of residence, n (%)			
Northeast	55 (9.2)	29 (12.2)	21 (11.6)
Midwest	259 (43.2)	103 (43.3)	81 (44.8)
South	175 (29.2)	66 (27.7)	47 (26.0)
West	111 (18.5)	40 (16.8)	32 (17.7)

a AHEI: Alternative Health Eating Index (ranging from 0 to 110, where a higher score reflects a healthier diet). The AHEI assigns ratings to foods and nutrients predictive of chronic disease (McCullough and Willett [36]). AHEI is created by a group of researchers at Harvard TH Chan School of Public Health as an alternative to the US Department of Agriculture’s Healthy Eating Index, which measures adherence to the federal Dietary Guidelines for Americans.

### Smartphone Data Characteristics

Data collection was completed in November 2021. In total, we received 40.0 GB of data from 181 participants, including 19.2 GB of accelerometer data, 0.6 GB of GPS data, and 0.01 GB of survey data. Each participant contributed data (at least 1 byte of data within the enrollment period) for an average of 7 out of 8 possible days (SD 2; median 8).

#### Smartphone-Based Survey Compliance

**Figure 2.**
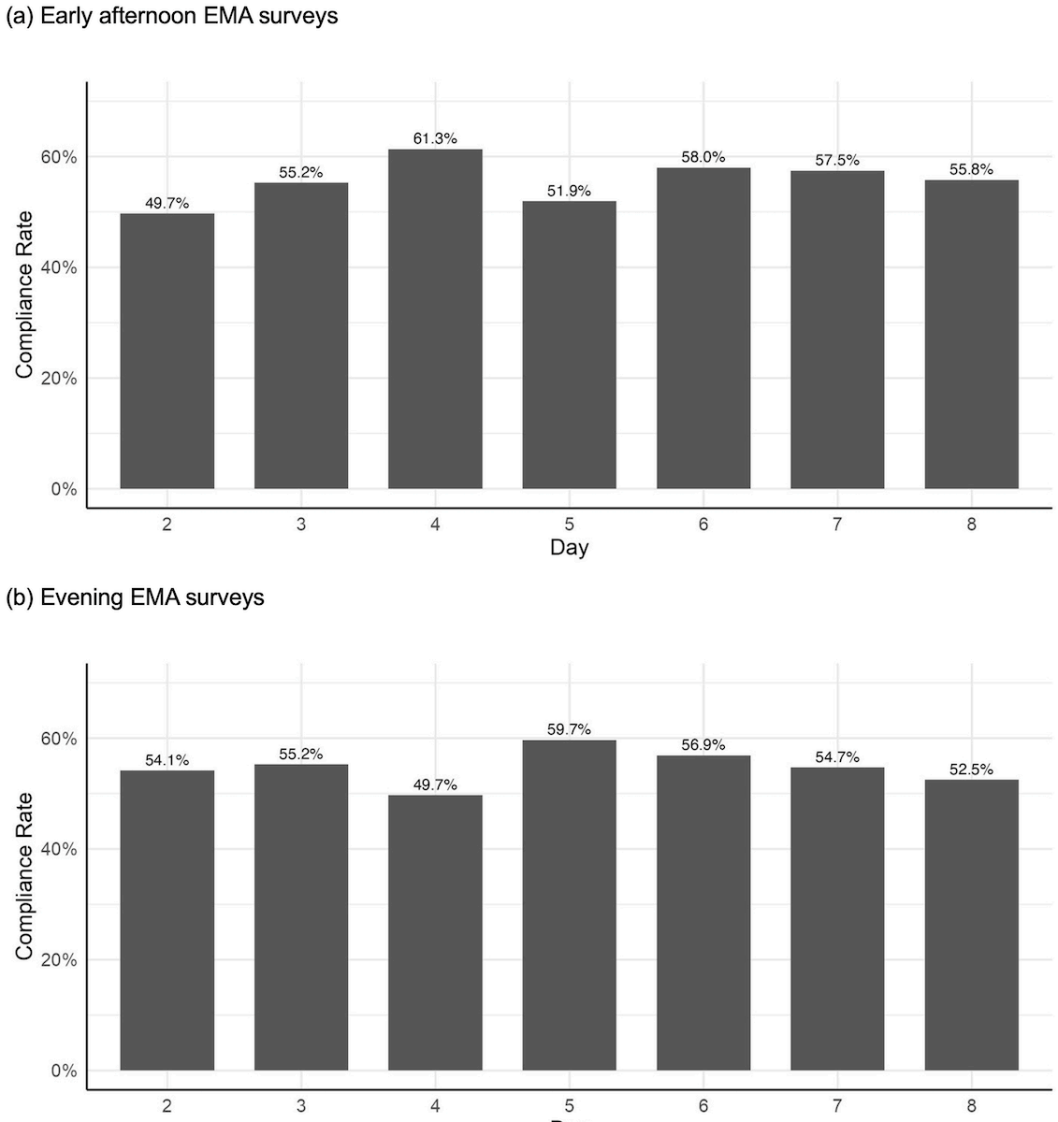
Daily completion with the EMA survey in the early afternoon (A) and evening (B) over the period of the study period (1 week) for the Beiwe Smartphone pilot study in the Nurses’ Health Study II (181 surveys sent each day per each EMA survey type). EMA: ecological momentary assessment.

Each afternoon and evening assessment was sent once a day for 181 participants for 7 days. A total of 181 participants completed 156 baseline surveys (86.2% response) at day 1. From days 2‐8, they responded to 705 early afternoon EMA prompts out of 1267 prompts sent (181 participants for 7 days; 55.6% response across all participant-days) and 693 evening EMA prompts out of 1267 prompts sent (181 participants for 7 days; 54.7% response across all participant-days). On average, the shorter surveys given with the early afternoon prompts took 1.5 minutes to complete (SD 8.3; median 0.8), while the longer surveys given with the evening prompts took 5.2 minutes (SD 5.3; median 4.3). The response rates to nonbranching items (ie, did not depend upon the answer of “Yes or No” in the previous question) within a submitted survey were 99% for early afternoon EMA (13 nonbranching items out of 21) and 96% for evening EMA surveys (30 nonbranching items out of 63). The daily response rates for the early afternoon and evening EMA surveys fluctuated between 50% and 60% across days 2-8 (mean 55.6%, SD 3.9% for early afternoon; mean 54.7%, SD 3.2% for evening; Figure 2). Each participant completed an average of 4 early afternoon prompts (SD 2; median 4) and 4 evening prompts (SD 2; median 4). Among the 181 participants, 136 (75.1%) completed >25%, 118 (65.2%) completed >50%, and 71 (39.2%) completed >75% of the early afternoon prompts. For evening prompts, 138 (76.2%), 116 (64.1%), and 69 (38.1%) participants completed >25%, >50%, and >75%, respectively (Table 2).

**Table 2.** Participant-level compliance of early afternoon and evening ecological momentary assessment surveys over the pilot study period (1 week) (N=181).

Variable	Overall, n (%)	
	Early afternoon survey	Evening survey
Participants completed >25% of prompts sent	136 (75.1)	138 (76.2)
Participants completed >50% of prompts sent	118 (65.2)	116 (64.1)
Participants completed >75% of prompts sent	71 (39.2)	69 (38.1)

#### Accelerometer and GPS Data Completeness

From 174 out of 181 participants, we collected an average of 11.8 valid hours (SD 5.6, median 12.9) of accelerometer data and 15.2 valid hours (SD 7.0, median 18.0) of GPS data per participant-day in days when any data were collected. Using the criterion of ≥10 valid hours for valid days (Table 3), we obtained 786 valid days (62.04% completeness) of accelerometer data and 731 valid days (57.70% completeness) of GPS data from 1267 potential data collection days (181 participants on days 2-8).

**Table 3.** Completeness of accelerometer and GPS data at the participant-day level over the pilot study period (N=1276^a^ total potential data collection days).

Participant day-level data completeness	Overall, n (%)	
	Accelerometer	GPS
Valid days	786 (62.04)	731 (57.70)
Invalid days	293 (23.13)	142 (11,21)
Noncollection days^b^	188 (14.84)	394 (31.10)

a Data included 7 participants who did not contribute any data.

b Noncollection days were defined as days without data. In this study, the primary reason for noncollection was attrition (ie, the app was deleted from a participant’s phone before the 7-day study period because the participant unenrolled from the substudy). Another reason was sensor noncollection, which could be due to a participant forgetting to charge their phone or disabling the GPS or a major update from the operating system causing the Beiwe app to malfunction temporarily, among other reasons.

**Figure 3.**
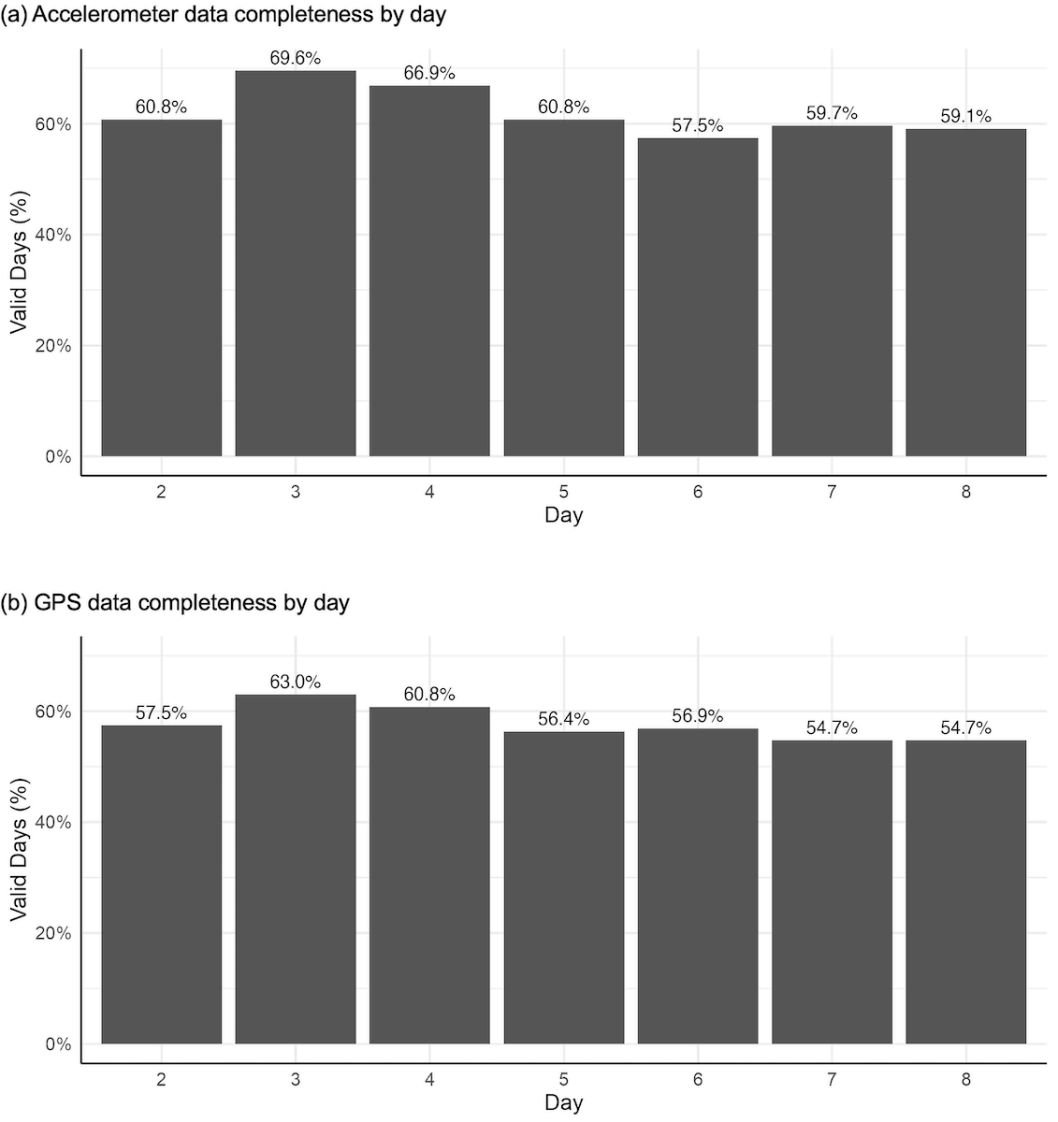
Daily completeness of accelerometer (A) and GPS (B) data over the pilot study period (N=1276 total potential data collection days). The statistics for day 1 were not included because the participants started at different times of the day, which meant that the validity criteria were not comparable.

Over time, completeness of the accelerometer and GPS data fluctuated between 54.7% and 69.6%, with accelerometer data having slightly higher percentages than GPS data (mean 62.0%, SD 4.5% for accelerometer; mean 57.7%, SD 3.1% for GPS; Figure 3).

For participant-level completeness, the average valid days for accelerometer and GPS data per participant were 4 for each parameter (SD 3; median 5). Among 181 participants, 145 (80.1%) had 25% valid days of accelerometer data, 112 (61.9%) had 50%, and 79 (43.6%) had 75%, while 125 (69.1%) had 25% valid days of GPS data, 105 (58.0%) had 50%, and 90 (49.7%) had 75% (Table 4).

**Table 4.** Participant-level data completeness of accelerometer and GPS data over the pilot study period (N=181).

Variable	Overall, n (%)	
	Accelerometer	GPS
Participants (N) who had:		
≥25% data days were valid days	145 (80.1)	125 (69.1)
≥50% data days were valid days	112 (61.9)	105 (58.0)
≥75% data days were valid days	79 (43.6)	90 (49.7)

### Feedback Survey

**Figure 4.**
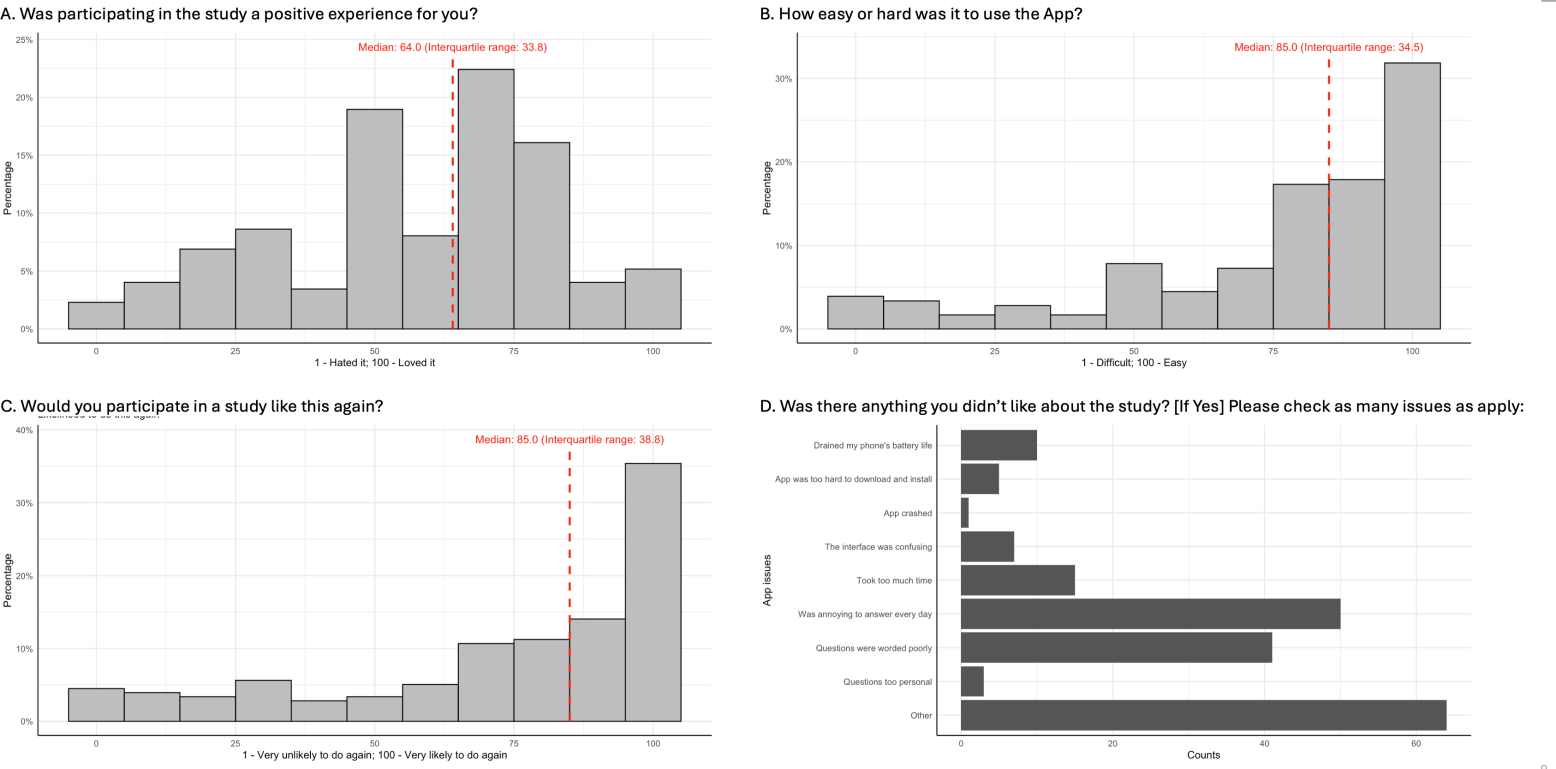
An overview of the distribution of answers to the questions in the feedback survey. (A) Whether the study was a positive experience. (B) The ease of use of the app. (C) Likelihood to participate again. (D) Things not liked about the study.

Participants reported an overall relatively “positive experience” (Figure 4A) with data collection (median 64.0 among 174 out of 181 participants, where 1=“I hated it” and 100=“I loved it”) and generally found the Beiwe app easy to use (median 85.0 among 179 out of 181 participants, where 1=“difficult” and 100=“easy”; Figure 4B). They also indicated a high likelihood of participating again (median 85.0 among 178 out of 181 participants, where 1=“Very unlikely” and 100=“Very likely”; Figure 4C). Despite this, across a list of potential concerns we provided, 2 main issues were selected with higher frequency (Figure 4D): the Substudy EMA prompt was “annoying to answer every day,” and the “questions were worded poorly.” Users also reported several major issues of the app, including “App was too hard to download and install” and “The interface was confusing.” Additional issues mentioned in the open-ended responses after selecting the “Other” answer included repetitive questions, unclear wording, the need for better notifications such as sound alerts, and lengthy completion times for some tasks (summarized in Multimedia Appendix 4). Users were also asked for open-ended feedback on problems faced during the study (Multimedia Appendix 5 summarized some commonly reported ones). Notably, we found the participants (19/181, 10.5%) who completed the feedback survey but not the baseline surveys mainly had issues including technical difficulties during app installation and registration, inability to reach the study staff for support, and lack of reminders.

## Discussion

### Principal Findings

This study examined adherence to a smartphone-based digital phenotyping protocol as part of a pilot study examining willingness to participate in an 8-day intensive measurement protocol to capture psychological well-being, health behaviors, and geolocation data among participants in the NHSII, a nationwide prospective cohort of women in the United States. Our findings indicated high compliance (86%) with the baseline survey and modest compliance (approximately 55% on average) with the daily EMA surveys, with no major differences evident based on the time at which the prompt was delivered (afternoon vs evening) or survey length (evening survey was longer). We also observed good levels of daily completeness (fluctuating between 55% and 70%) of passive sensing data (accelerometer and GPS) using the criterion of 10 valid hours per day. Even with overall modest survey compliance, feedback survey results indicated that participants found the app user-friendly and were willing to engage in similar future studies. However, they also highlighted issues, such as the need for improved survey question wording and user fatigue from repetitive daily surveys. Overall, our evaluation provides valuable insights and lessons for future studies aimed at incorporating smartphone-based digital phenotyping in large prospective cohorts with participants who more often engage in surveys and periodic assessments than in intensive measurement bursts.

Compared with previous smartphone-based EMA studies that collected data on psychological well-being, our day-level compliance statistics were lower overall. For example, a 2021 systematic review of smartphone-based EMA studies that focused on psychological well-being [38] reported an average compliance rate of 71.6% based on all EMA prompts in each study across 53 studies with a median duration of 7 days. However, these studies often recruited convenience samples and provided compensation to participants. In addition, their participants were often younger (mean 30.8, SD 10.5 years) than our participants (mean 67.8, SD 3.9 years), who may encounter less technological barriers. Furthermore, these studies also represented more of a general population, likely more flexible in survey schedules than our population who were nurses and nursing professionals at baseline. Interestingly, in our study, compliance for the early afternoon and evening EMA surveys varied between 50% and 60% and showed no signs of user fatigue (ie, compliance rates decreased over time). In addition, we found no substantial differences in compliance rates for individual questions in either early afternoon or evening EMA surveys. This is somewhat surprising given that the most common complaint in the feedback survey was that the EMA survey was “boring to answer every day.” This may be due in part to conducting this study among a cohort of participants who have been engaged and active in the study for many years.

When we examined survey compliance at the participant level, we found that 32 out of 181 participants were noncompliant (did not submit any early afternoon or evening survey). We therefore speculate that the lower than average compliance with the daily EMA survey may be due to the initial technical difficulties (another common complaint found in the feedback survey) that users encountered in registering and setting up the Beiwe app, which led to early dropout. Therefore, we suggest that future studies ensure timely and adequate technical support during app installation, registration, and throughout the study. This is particularly crucial when the study contains a population that may face higher usability barriers for smartphone apps, such as the older adults in our pilot who may have varying levels of digital literacy [39].

Moreover, according to the feedback survey, participants suggested smartphone EMA survey design ideas to potentially improve their compliance with the schedule, such as daily reminders and notification enhancements with visual or sensory triggers such as icons or sounds, as well as simplifying the layout of the app. We suggest incorporating these functionalities in future studies, particularly in occupational populations with busy work schedules, such as nurses and nursing professionals in our cohort.

Additionally, another 2021 systematic review and meta-analysis [40] found a negative association between the number of items per prompt and EMA compliance in nonclinical studies assessing self-reported health behaviors and psychological constructs in adults. Consequently, our surveys might impose a higher user burden and result in quicker user fatigue than those with fewer questions (eg, asking “how is your current well-being” 3 times a day through established constructs such as Life Orientation Test—Revised and PANAS-X, while most studies previously inquired this through 1 or 2 questions) [41]. Future research should explore strategies to mitigate survey fatigue, such as alternating between truncated and full versions of the EMA survey daily or adopting an intensive longitudinal approach (ie, space out multiple week-long surveys within a year or over several years) [40].

The completeness of our accelerometer and GPS data is slightly lower than that of previous smartphone-based studies that collected similar types of data [17,38,42]. However, this was not unexpected given few studies have simultaneously collected smartphone-based psychological well-being and health behavior data [43-45], which may place higher user burden. From our observations, one of the main reasons for data incompleteness in the passive sensing data was attrition (ie, the app was deleted from a participant’s phone before the 7-day study completion date because the participant was unenrolled from the substudy). Besides, to minimize failures in the collection of smartphone passive sensing data, we instructed participants at the start of the data collection period to always carry their smartphones with them. Nonetheless, data noncollection or device noncompliance may still be higher than in similar studies due to the older age of our participants, who may be less likely to carry their smartphones everywhere or keep smartphones on them and charged, compared with the younger populations in these studies [43,44]. This issue was further compounded by our noncontinuous sampling strategy for passive sensing data and the tendency of phone operating systems to disable sensor access when the phone is inactive for extended periods, such as during sleep.

In addition, data collection may also be disrupted by major updates of phone operating system, which may cause the Beiwe app to malfunction temporarily. We recommend that future studies experiment with more advanced passive sensing schemes, such as turning sensors on and off based on the participants’ movement status (ie, moving or staying sedentary) rather than a fixed frequency [46], to further conserve batteries while improving the data quality of geolocations and activity tracking.

Furthermore, we found that the completeness of the passive sensing data over the study period was overall higher than EMA survey compliance rate. This indicates that passive sensing data collection may impose a lower burden than smartphone-based surveys and can be extended longer, which is desirable as a 1-week period may not be sufficient for capturing the usual activity patterns of study participants [17,47]. Future studies should consider varying the data collection duration for each data stream instead of maintaining a fixed period for all or reducing the frequency of smartphone surveys (eg, from daily to every other day).

Collecting highly resolved smartphone-based data on psychological well-being and physical activity will facilitate novel investigations of their relationships at finer temporal resolutions, such as daily or diurnal levels. For instance, we can examine whether a stressful event and strategies used to regulate one’s affective states in this context, as reported by the EMA in the last hour, are associated with physical activity levels in the subsequent (or prior) hours. Additionally, we can analyze whether daily positive or negative affect correlates with physical activity on the same or the following day. Furthermore, linking location-based behavior data to environmental factors allows examining the relationship between psychological well-being, health behaviors, and momentary environmental exposures. For example, one could examine whether GPS-based greenspace exposure in the previous hour impacts EMA-reported positive affect. In another example, one can explore momentary associations between GPS-based temperature or PM_2.5_ exposure and smartphone-assessed physical activity [9,48-52].

### Strengths and Limitations

Our study has certain limitations. Although our walking recognition algorithm accounts for missing data due to noncontinuous sampling, we may still miss some forms of physical activity due to participants not carrying their smartphones while engaging in these types of activities, which could impact the representativeness of the collected data. Our GPS data may have additional missing data due to environmental conditions (eg, being indoors and heavy tree coverage that can interfere with GPS satellite signals). Additionally, the 1-week data collection period may not capture variability in physical activity and mobility over longer durations, such as seasonal changes [17,53]. The EMA-prompting scheme, administered twice daily, may also fail to capture within-day variations in some experiences, such as positive and negative affect [54]. Furthermore, the NHSII study’s homogeneous population—predominantly White, middle-aged and older women who were nurses at the 1989 baseline and have maintained high engagement rates with the cohort over long follow-up periods [18]—may limit the generalizability of our findings regarding device compliance and feasibility of embedding an EMA study within a larger cohort study with participants who generally engage in periodic surveys. Nonetheless, all epidemiologic cohorts focus on compliant participants, and thus our results are informative, especially for other cohorts that may seek to embed smartphone data collection into existing studies.

This study has several strengths. This is one of the first studies to comprehensively examine adherence to an intensive smartphone measurement burst protocol in a large, nationwide prospective cohort of middle-aged and older women. Unlike previous studies, we used multiple metrics to assess compliance with daily EMA surveys over an 8-day period and the completeness of passive sensing data during the same time frame. Our findings offer crucial, detailed insights into the feasibility of and participation in an embedded smartphone-based digital phenotyping protocol into epidemiological cohorts, amidst growing interest in this area [55]. Additionally, the study incorporated feedback survey data, providing subjective and individualized insights that complement objective metrics and help explain trends observed in adherence. This feedback also highlights potential improvements in future study designs and app functionalities.

### Conclusions

This pilot study investigated adherence to a smartphone-based data collection protocol embedded within an ongoing cohort study, assessing willingness to participate in an 8-day intensive measurement burst to collect data on psychological well-being, physical activity, and mobility. Our findings suggest modest compliance with smartphone EMA surveys and good completeness of passive sensing data is possible. Adherence remained stable over the 1-week period. Notably, participants expressed a strong interest in participating in similar future studies, according to the feedback survey. These findings have major implications for future research aimed at intensively measuring psychological well-being and physical activity over short periods via smartphones. Smartphone-based digital phenotyping allows researchers to use devices that participants already have and are familiar with for data collection, rather than mailing out wearables, which could be particularly important when seeking to recruit large numbers of participants from established epidemiological cohorts. Overall, these methodologies appear promising for advancing epidemiological research on the psychological and behavioral determinants of health.

## Supplementary material

10.2196/71375 Multimedia Appendix 1 Screenshots of the customized Beiwe app used in the Beiwe Smartphone Substudy of Nurses’ Health Study II.

10.2196/71375 Multimedia Appendix 2 List of survey questions and answer choices delivered by the Beiwe app.

10.2196/71375 Multimedia Appendix 3 Demographic characteristics of the Beiwe Smartphone Substudy of Nurses’ Health Study II cohort by phone operating systems.

10.2196/71375 Multimedia Appendix 4 A summary of comments participants made to improve the participant experience.

10.2196/71375 Multimedia Appendix 5 A summary of comments participants made on issues of the app other than the options given.

## Abbreviations

AHEI: Alternative Healthy Eating Index
CONSORT: Consolidated Standards of Reporting Trials
EMA: ecological momentary assessment
NHSII: Nurses’ Health Study II
PANAS-X: Positive and Negative Affect Schedule—Expanded Form
