# Supplementary material for: Measuring Psychological Well-Being and Behaviors Using Smartphone-Based Digital Phenotyping: An Intensive Longitudinal Observational mHealth Pilot Study Embedded in a Prospective Cohort of Women
Source: JMIR Mhealth Uhealth. 2025 Sep 3;13:e71375. doi: 10.2196/71375 (PMC12407220; doi:10.2196/71375)
Supplement: Multimedia Appendix 1 [file mhealth-v13-e71375-s001.docx]

| 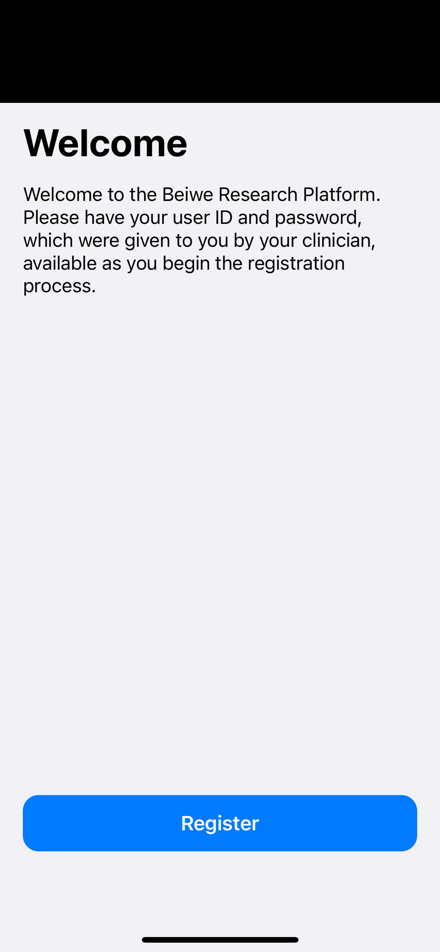 | 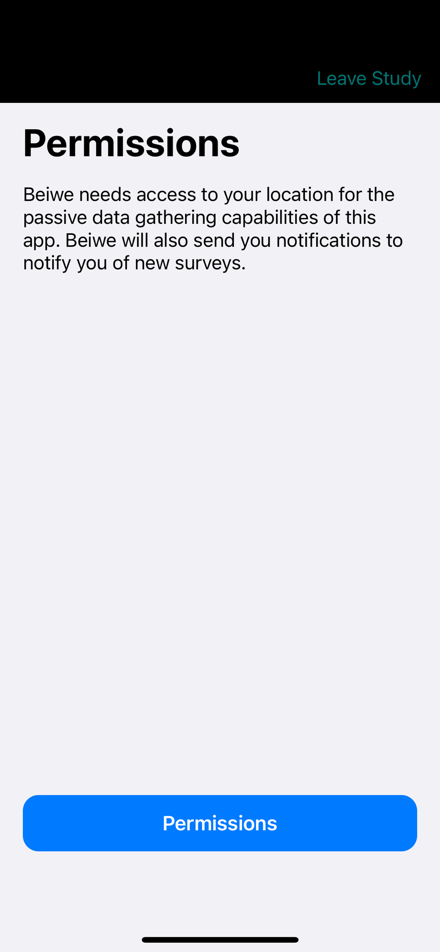 | 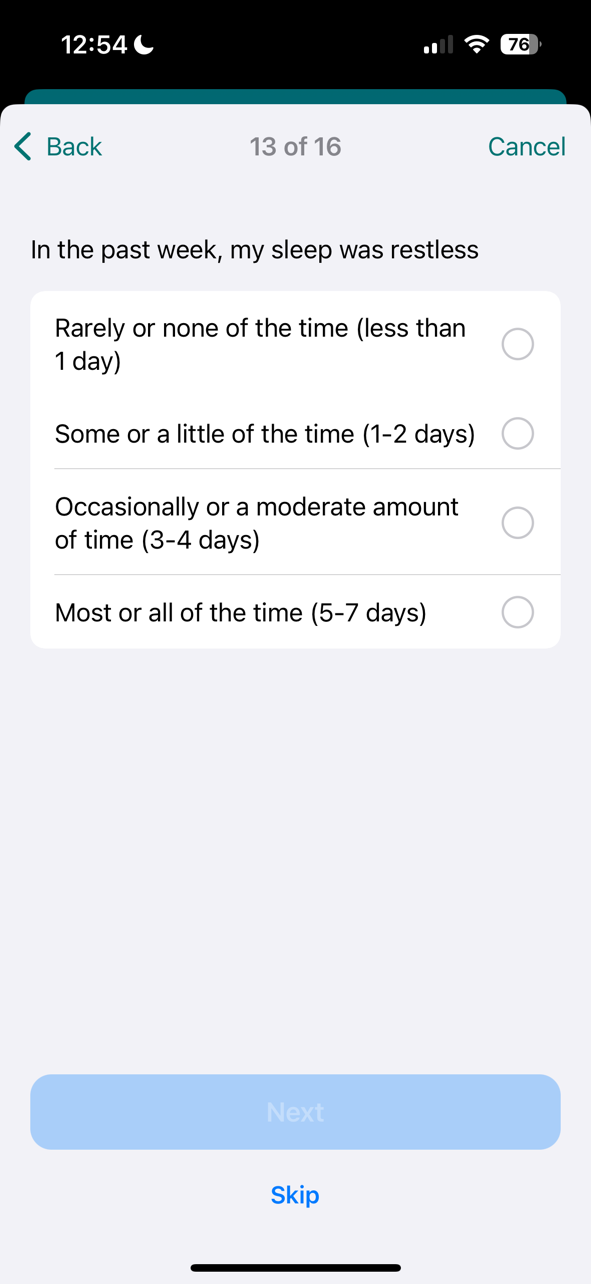 |
| --- | --- | --- |

**Multimedia Appendix 1.** Screenshots of the customized Beiwe app used in the Beiwe Smartphone Substudy of Nurses’ Health Study II (NHS2).
