# Supplementary material for: Measuring Psychological Well-Being and Behaviors Using Smartphone-Based Digital Phenotyping: An Intensive Longitudinal Observational mHealth Pilot Study Embedded in a Prospective Cohort of Women
Source: JMIR Mhealth Uhealth. 2025 Sep 3;13:e71375. doi: 10.2196/71375 (PMC12407220; doi:10.2196/71375)
Supplement: Multimedia Appendix 2 [file mhealth-v13-e71375-s002.docx]

**Multimedia Appendix 2.** List of survey questions and answer choices delivered by the Beiwe App.

***Baseline Survey***

| **Survey Constructs** | **Survey Questions** | **Answer Choices** |
| --- | --- | --- |
| **Revised Life Orientation Test (LOT-R)** | 1. In uncertain times, I usually expect the best  2. If something can go wrong for me, it will  3. I’m always optimistic about my future  4. I hardly ever expect things to go my way  5. I rarely count on good things happening to me  6. Overall, I expect more good things to happen to me than bad | - Strongly Disagree - Disagree - Neutral - Agree - Strongly Agree |
| **Center for Epidemiological Studies Depression (CES-D)** | 1. I was bothered by things that usually don’t bother me  2. I had trouble keeping my mind on what I was doing  3. I felt depressed  4. I felt that everything I did was an effort  5. I felt hopeful about the future  6. I felt fearful  7. My sleep was restless  8. I was happy  9. I felt lonely  10. I could not “get going” | - Rarely or none of the time (less than 1 day) - Some or a little of the time (1‐2 days) - Occasionally or a moderate amount of time (3‐4 days) - Most or all of the time (5‐7 days) |

Scheier, M. F., Carver, C. S., & Bridges, M. W. (1994). Distinguishing optimism from neuroticism (and trait anxiety, self-mastery, and self-esteem): A reevaluation of the Life Orientation Test. Journal of Personality and Social Psychology, 67(6), 1063-1078.

Radloff, L. S. (1977). The CES-D scale: A self report depression scale for research in the general population. Applied Psychological Measurements, 1, 385-401.

***Early Afternoon EMA Survey***

| **Survey Constructs** | **Survey Questions** | **Answer Choices** |
| --- | --- | --- |
| **PANAS-X** | 'Right now; I feel happy' | - Strongly Disagree - Disagree - Neutral - Agree - Strongly Agree |
|  | 'Right now; I feel determined' |  |
|  | 'Right now; I feel enthusiastic' |  |
|  | 'Right now; I feel curious' |  |
|  | 'Right now; I feel sad' |  |
|  | 'Right now; I feel afraid' |  |
|  | 'Right now; I feel angry' |  |
|  | 'Right now; I feel lonely' |  |
|  | 'Right now; I feel nervous' |  |
|  | 'Right now; I feel worried' |  |
| **Emotion regulation & coping strategies** | Did anything stressful occur in the past hour? | - No - Yes |
|  | [If Yes] How would you describe this stressful event? If more than one event happened; please pick the most significant event. [Check One] | - Argument/conflict/disagreement - Financial event - Work-related event - Health event or accident - Event that happened to others - Traffic or transportation event - Other |
|  | How stressful was this for you? | - Not at all - A little - Somewhat - Very |
|  | How did you respond to this event? Please consider how much you agree with each statement: "I thought about the situation in a different way." | - Strongly disagree - Disagree - Neutral - Agree - Strongly agree |
|  | How did you respond to this event? Please consider how much you agree with each statement: "I controlled my emotions by not showing them." |  |
|  | How did you respond to this event? Please consider how much you agree with each statement: "I accepted the situation and/or my emotions." |  |
|  | How did you respond to this event? Please consider how much you agree with each statement: "I thought over and over again about the situation and my feelings." |  |
|  | How did you respond to this event? Please consider how much you agree with each statement: "I made a plan to make the situation better." |  |
|  | How did you respond to this event? Please consider how much you agree with each statement: "I expressed gratitude towards someone; myself; or a life circumstance (for example; health or family)." |  |

***Evening EMA Survey***

| **Survey Constructs** | **Survey Questions** | **Answer Choices** |
| --- | --- | --- |
| **Social Contexts** | Who is with you right now [Check all that apply] | - Nobody - Partner/Spouse - Family - Friends - Co-workers - Pet - Other |
| **Physical Contexts** | Where are you right now? [Select one] | - Home - Work - School - Another Person's Home - Vehicle - Outside - Other |
| **PANAS-X** | Today; did you feel happy? | [No;Yes] |
|  | When did you feel happy today? [Check all that apply] | [Midnight-5:59a;6:00a-11:59a;Noon-5:59p;6:00p-11:59p] |
|  | Today; did you feel determined? | [No;Yes] |
|  | When did you feel determined today? [Check all that apply] | [Midnight-5:59a;6:00a-11:59a;Noon-5:59p;6:00p-11:59p] |
|  | Today; did you feel enthusiastic? | [No;Yes] |
|  | When did you feel enthusiastic today? [Check all that apply] | [Midnight-5:59a;6:00a-11:59a;Noon-5:59p;6:00p-11:59p] |
|  | Today; did you feel curious? | [No;Yes] |
|  | When did you feel curious today? [Check all that apply] | [Midnight-5:59a;6:00a-11:59a;Noon-5:59p;6:00p-11:59p] |
|  | Today; did you feel grateful? | [No;Yes] |
|  | When did you feel grateful today? [Check all that apply] | [Midnight-5:59a;6:00a-11:59a;Noon-5:59p;6:00p-11:59p] |
|  | Today; did you feel sad? | [No;Yes] |
|  | When did you feel sad today? [Check all that apply] | [Midnight-5:59a;6:00a-11:59a;Noon-5:59p;6:00p-11:59p] |
|  | Today; did you feel afraid? | [No;Yes] |
|  | When were you afraid today? [Check all that apply] | [Midnight-5:59a;6:00a-11:59a;Noon-5:59p;6:00p-11:59p] |
|  | Today; did you feel angry? | [No;Yes] |
|  | When did you feel angry today? [Check all that apply] | [Midnight-5:59a;6:00a-11:59a;Noon-5:59p;6:00p-11:59p] |
|  | Today; did you feel lonely? | [No;Yes] |
|  | When did you feel lonely today? [Check all that apply] | [Midnight-5:59a;6:00p-11:59p;Noon-5:59p;6:00p-11:59p] |
|  | Today; did you feel nervous? | [No;Yes] |
|  | When did you feel nervous today? [Check all that apply] | [Midnight-5:59a;6:00a-11:59a;Noon-5:59p;6:00p-11:59p] |
|  | Today; did you feel worried? | [No;Yes] |
|  | When did you feel worried today? [Check all that apply] | [Midnight-5:59a;6:00a-11:59a;Noon-5:59p;6:00p-11:59p] |
| **Emotion Regulation** | What was your most intense positive emotion today? [Check one] | [Happy;Determined;Enthusiastic;Curious;Grateful;None Experienced] |
|  | The next few questions refer to your most intense positive emotion today. How did you respond when feeling this emotion? Think about the situation that gave rise to this emotion; and consider how much you agree with the following statement: "I reveled in the moment and concentrated on how good I felt." | [Strongly disagree;Disagree;Neutral;Agree;Strongly Agree] |
|  | How did you respond when feeling this emotion? Think about the situation that gave rise to this emotion; and consider how much you agree with the following statement: "I expressed gratitude towards someone; myself; or a life circumstance (for example; health or family)." | [Strongly disagree;Disagree;Neutral;Agree;Strongly Agree] |
|  | How did you respond when feeling this emotion? Think about the situation that gave rise to this emotion; and consider how much you agree with the following statement: "I thought about all the good things that were happening in my life." | [Strongly disagree;Disagree;Neutral;Agree;Strongly Agree] |
|  | In response to this emotion; did you change your diet to celebrate? | [Yes: I increased how much I ate;Yes: I decreased how much I ate;No: I did not change how much I ate] |
|  | Which was your most intense negative emotion today? | [Sad;Afraid;Angry;Lonely;Nervous;Worried;None Experienced] |
|  | The next few questions refer to your most intense negative emotion today. How did you respond when feeling this emotion? Think about the situation that gave rise to this emotion; and consider how much you agree with the following statement: "I thought about the situation in a different way." | [Strongly disagree;Disagree;Neutral;Agree;Strongly Agree] |
|  | How did you respond when feeling this emotion? Think about the situation that gave rise to this emotion; and consider how much you agree with the following statement: "I controlled my emotions by not showing them." | [Strongly disagree;Disagree;Neutral;Agree;Strongly Agree] |
|  | How did you respond when feeling this emotion? Think about the situation that gave rise to this emotion; and consider how much you agree with the following statement: "I accepted the situation and/or my emotions." | [Strongly disagree;Disagree;Neutral;Agree;Strongly Agree] |
|  | How did you respond when feeling this emotion? Think about the situation that gave rise to this emotion; and consider how much you agree with the following statement: "I thought over and over again about the situation and my feelings." | [Strongly disagree;Disagree;Neutral;Agree;Strongly Agree] |
|  | How did you respond when feeling this emotion? Think about the situation that gave rise to this emotion; and consider how much you agree with the following statement: "I made a plan to make the situation better." | [Strongly disagree;Disagree;Neutral;Agree;Strongly Agree] |
|  | How did you respond when feeling this emotion? Think about the situation that gave rise to this emotion; and consider how much you agree with the following statement: "I expressed gratitude towards someone; myself; or a life circumstance (for example; health or family)." | [Strongly disagree;Disagree;Neutral;Agree;Strongly Agree] |
|  | Did you change your diet in response to this emotion? | [Yes: I increased how much I ate;Yes: I decreased how much I ate;No: I did not change how much I ate] |
|  | In response to this emotion; did you change your diet as a way to comfort yourself? | [No;Yes] |
|  | In response to this emotion; did you change your diet in order to distract you from thinking about something unpleasant? | [No;Yes] |
| **Response to Stressful Events and Positive Experience** | In the past 24 hours did you have an argument or disagreement with anyone? (E.g.; with a family member) | [No;Yes] |
|  | How stressful was this for you? | [Not at all;A little;Somewhat;Very] |
|  | In the past 24 hours did anything happen (other than what you have already mentioned) that you could have argued or disagreed about; but you decided to let it pass? | [No;Yes] |
|  | How stressful was this for you? | [Not at all;A little;Somewhat;Very] |
|  | In the past 24 hours did anything happen in your workplace or volunteer setting (other than what you have already mentioned) that most people would consider stressful? | [No;Yes] |
|  | How stressful was this for you? | [Not at all;A little;Somewhat;Very] |
|  | In the past 24 hours did anything happen at home (other than what you have already mentioned) that most people would consider stressful? | [No;Yes] |
|  | How stressful was this for you? | [Not at all;A little;Somewhat;Very] |
|  | In the past 24 hours did anything happen to a close friend or relative (other than what you have already mentioned) that turned out to be stressful for you? | [No;Yes] |
|  | How stressful was this for you? | [Not at all;A little;Somewhat;Very] |
|  | In the past 24 hours did you have any positive experiences with anyone in your family? | [No;Yes] |
|  | How positive would you rate the experience? | [Not at all;A little;Somewhat;Very] |
|  | In the past 24 hours did you have any positive experiences with other people outside your family? | [No;Yes] |
|  | How positive would you rate the experience? | [Not at all;A little;Somewhat;Very] |
|  | In the past 24 hours did you have any positive experiences in your workplace or volunteer setting? | [No;Yes] |
|  | How positive would you rate the experience? | [Not at all;A little;Somewhat;Very] |
|  | In the past 24 hours did a close friend or relative have any positive experiences that affected you? | [No;Yes] |
|  | How positive would you rate the experience? | [Not at all;A little;Somewhat;Very] |
| **Sleep** | What time did you fall asleep last night? [Pick the time closest to when you fell asleep] | [5:00p;5:30p;6:00p;6:30p;7:00p;7:30p;8:00p;8:30p;9:00p;9:30p;10:00p;10:30p;11:00p;11:30p;12:00a;12:30a;1:00a;1:30a;2:00a;2:30a;3:00a;3:30a;4:00a;4:30a;5:00a;5:30a;6:00a;6:30a;7:00a;7:30a;8:00a;8:30a;9:00a;9:30a;10:00a;10:30a;11:00a;11:30a;12:00p;12:30p;1:00p;1:30p;2:00p;2:30p;3:00p;3:30p;4:00p;4:30p] |
|  | What time did you wake up this morning? [Pick the time closest to when you woke up] | [1:00a;1:30a;2:00a;2:30a;3:00a;3:30a;4:00a;4:30a;5:00a;5:30a;6:00a;6:30a;7:00a;7:30a;8:00a;8:30a;9:00a;9:30a;10:00a;10:30a;11:00a;11:30a;12:00p;12:30p;1:00p;1:30p;2:00p;2:30p;3:00p;3:30p;4:00p;4:30p;5:00p;5:30p;6:00p;6:30p;7:00p;7:30p;8:00p;8:30p;9:00p;9:30p;10:00p;10:30p] |
|  | Please rate the quality of your sleep last night. | [Very Bad;Fairly Bad;Fairly Good;Very Good] |
| **Prescription Medication** | Did you take any of the following prescription medications yesterday? [Check as many as apply] | [Antidepressant medication (for any reason);Antianxiety medication;Sleep medication;None of the above] |
| **Satisfaction with Life** | How satisfied are you with your life at this moment? [1 being very unsatisfied; 5 being very satisfied] | min = 1; max = 5 |

***Feedback Survey***

| **Survey Questions** | **Answer Choices** |
| --- | --- |
| Was participating in the study a positive experience for you? | 1 – Hated it; 100 – Loved it |
| Are there ways we could make the experience better? | ** Participants could add comments* |
| How easy or hard was it to use the App? | 1 – Difficult; 100 – Easy |
| Ability to complete the protocol (a full week of sampling) | [Mostly; No; Yes] |
| Was there anything you didn’t like about the study?  [If Yes] Please check as many issues as apply: | ["Drained my phone's battery life",  "App was too hard to download and install",  "App crashed",  "The interface was confusing",  "Took too much time",  "Was annoying to answer every day",  "Questions were worded poorly",  "Questions too personal",  "Other"]  ** For the option “other”, participants could add comments* |
| Would you participate in a study like this again? | 1 – Very unlikely to do again; 100 – Very likely to do again |
