# Supplementary material for: Measuring Psychological Well-Being and Behaviors Using Smartphone-Based Digital Phenotyping: An Intensive Longitudinal Observational mHealth Pilot Study Embedded in a Prospective Cohort of Women
Source: JMIR Mhealth Uhealth. 2025 Sep 3;13:e71375. doi: 10.2196/71375 (PMC12407220; doi:10.2196/71375)
Supplement: Multimedia Appendix 3 [file mhealth-v13-e71375-s003.docx]

**Multimedia Appendix 3.** Demographic characteristics of the Beiwe Smartphone Substudy of Nurses’ Health Study II cohort by phone operating systems.

|  | **Beiwe Substudy Participants - Registered and Downloaded the App** | **Participants with iOS phones** | **Participant with Android phones** |
| --- | --- | --- | --- |
| **Variable** | **n = 181** | **n = 152** | **n = 29** |
| **Age (years), mean (SD)** | 67.8 (3.9) | 67.9 (3.9) | 67.7 (3.9) |
| **Race, n (%)** |  |  |  |
| White | 178 (98.3) | 149 (98.0) | 29 (100.0) |
| Black | 0 (0.0) | 0 (0.0) | 0 (0.0) |
| Other/more than one race | 3 (1.7) | 3 (2.0) | 0 (0.0) |
| **Husband’s highest level of education, n (%)** |  |  |  |
| High school or less | 51 (28.2) | 39 (25.7) | 12 (41.4) |
| More than high school | 130 (71.8) | 113 (74.3) | 17 (58.6) |
| **Marital status, n (%)** |  |  |  |
| Married | 148 (81.8) | 123 (80.9) | 25 (86.2) |
| **Smoking Status, n (%)** |  |  |  |
| Current | 5 (2.8) | 5 (3.3) | 0 (0.0) |
| Former | 70 (38.7) | 56 (36.8) | 14 (48.3) |
| Never | 106 (58.6) | 91 (59.9) | 15 (51.7) |
| **BMI (kg/m^2^), mean (SD)** | 27.0 (6.4) | 27.0 (6.4) | 27.2 (6.4) |
| **AHEI score^a^, mean (SD)** | 70.0 (12.4) | 69.9 (12.8) | 71.1 (9.7) |
| **Region of Residence, n (%)** |  |  |  |
| Northeast | 21 (11.6) | 17 (11.2) | 4 (13.8) |
| Midwest | 81 (44.8) | 70 (46.1) | 11 (37.9) |
| South | 47 (26.0) | 39 (25.7) | 8 (27.6) |
| West | 32 (17.7) | 26 (17.1) | 6 (20.7) |
