# Supplementary material for: Measuring Psychological Well-Being and Behaviors Using Smartphone-Based Digital Phenotyping: An Intensive Longitudinal Observational mHealth Pilot Study Embedded in a Prospective Cohort of Women
Source: JMIR Mhealth Uhealth. 2025 Sep 3;13:e71375. doi: 10.2196/71375 (PMC12407220; doi:10.2196/71375)
Supplement: Multimedia Appendix 4 [file mhealth-v13-e71375-s004.docx]

**Multimedia Appendix 4.** A summary of comments participants made to improve the participant experience.

| **Comment categories** | **Summary of comments made** |
| --- | --- |
| Technical Difficulties | - Many users experienced glitches, such as issues with the cursor not selecting answers, difficulty downloading the App, problems with notifications, and difficulties logging in or accessing the App. |
| Survey Design and Content | - Repetitiveness: A common complaint was the repetitive nature of the questions. Users suggested using varied language or reducing the frequency of surveys. - Question Clarity and Relevance: Some found the questions vague, too generic, or not capturing their feelings accurately. Users recommended including more diverse response options, such as "other" or "n/a," and providing context for questions. - Timing of Surveys: Users suggested consistent timing for surveys and ensuring the questions are relevant to the time of day they are answered. - Inclusion of Additional Options: Suggestions included asking about pain, allowing narrative responses, and providing more adjectives for choices. |
| User Experience and Interface | - Improved Notifications: Users requested better notifications, such as sound alerts or more visible indicators, and consistent reminders. - Ease of Use: The App should be more user-friendly, with clear instructions, easier log-in methods (like face recognition), and functioning links. - Survey Duration: Users noted that the surveys sometimes extended beyond the stated duration, leading to confusion and frustration. - Personalization and Contextual Understanding: Users felt that understanding their background or current situation could help interpret their responses better. They suggested that the App could benefit from collecting some background information or allowing users to provide comments for context. |
| Communication and Support | - There were issues with reaching support staff, with full mailboxes and unanswered emails. Improving support and ensuring users can easily get help is crucial. |
| Privacy and Transparency | - Some users expressed concerns about what data the App was collecting and the purpose of the study. Clear communication about the study's objectives and data usage is important. |
