# Supplementary material for: Measuring Psychological Well-Being and Behaviors Using Smartphone-Based Digital Phenotyping: An Intensive Longitudinal Observational mHealth Pilot Study Embedded in a Prospective Cohort of Women
Source: JMIR Mhealth Uhealth. 2025 Sep 3;13:e71375. doi: 10.2196/71375 (PMC12407220; doi:10.2196/71375)
Supplement: Multimedia Appendix 5 [file mhealth-v13-e71375-s005.docx]

**Multimedia Appendix 5.** A summary of comments participants made on issues of the app other than the options given.

| **Comment categories** | **Summary of comments made** |
| --- | --- |
| Technical Issues and Accessibility | - Difficulty accessing the App and issues with passwords not working. - App not functioning correctly on various devices (e.g., issues on Samsung phones, iPhone 6 screen too small). - The App not loading or problems with installation. |
| User Interface and Experience | - Repetitive questioning, leading to user boredom and dread. - Interface issues, such as difficulty selecting options and navigating the App. - Lack of clear instructions or confusion about how to use the App and interpret questions. - The absence of audio notifications for new surveys. |
| Content and Structure of Surveys | - Questions perceived as too repetitive, irrelevant, or lacking depth. - The structure of the questions not aligning with the user's daily routine (e.g., questions about bedtime appearing too late). - Limited response options not adequately capturing users' feelings or situations. - The need for more neutral answer options and a comment section for additional context. - Confusion about how to answer based on the timing of the survey (e.g., answering in the morning about the entire day). |
| Privacy and Trust Concerns | - Discomfort with the App tracking activity and location. - Concerns about data privacy and the use of personal information. |
| Study Management and Communication | - The study going beyond the initially stated duration without clear communication. - Difficulty contacting support staff or getting assistance with issues. - Uncertainty about when the study was complete or when to stop participating. |
